# Tobacco use trends among youth in Saudi Arabia: 2007–2022

**DOI:** 10.3389/fpubh.2025.1608394

**Published:** 2025-07-02

**Authors:** Sarah S. Monshi, Eman D. El Desouky, Shaikha K. Aldukhail, Abdulmohsen H. Al-Zalabani, Mohammed M. Alqahtani, Mervat M. El Dalatony, Mosa A. Shubayr, Ahmed A. Elkhobby, Mohammed S. Aldossary

**Affiliations:** ^1^Department of Health Administration and Hospitals, College of Public Health and Health Informatics, Umm Al Qura University, Makkah, Saudi Arabia; ^2^General Directorate of Research and Studies, Ministry of Health, Riyadh, Saudi Arabia; ^3^Department of Preventive Dental Sciences, College of Dentistry, Princess Nourah Bint Abdulrahman University, Riyadh, Saudi Arabia; ^4^Department of Family and Community Medicine, College of Medicine, Taibah University, Madinah, Saudi Arabia; ^5^Department of Respiratory Therapy, College of Applied Medical Sciences, King Saud bin Abdulaziz University for Health Sciences, Riyadh, Saudi Arabia; ^6^King Abdullah International Medical Research Center, Riyadh, Saudi Arabia; ^7^Department of Respiratory Services, King Abdulaziz Medical City, Ministry of National Guard Health Affairs, Riyadh, Saudi Arabia; ^8^Department of Public Health and Community Medicine, Faculty of Medicine, Menoufia University, Shebin El Kom, Egypt; ^9^Department of Preventive Dental Sciences, College of Dentistry, Jazan University, Jazan, Saudi Arabia; ^10^Tobacco Control Program, Ministry of Health, Riyadh, Saudi Arabia

**Keywords:** tobacco use, smoking, shisha, adolescent health, youth, Saudi Arabia

## Abstract

**Introduction:**

Tobacco use is a major global public health challenge, causing millions of deaths annually. In Saudi Arabia, increasing adult smoking rates highlight the need for improved tobacco control measures.

**Aim:**

This study examined trends in tobacco use among youth in Saudi Arabia from 2007 to 2022, evaluating shifts in the determinants of tobacco use and the effectiveness of control measures over time.

**Methods:**

We analyzed three waves of the Global Youth Tobacco Survey (GYTS) conducted in Saudi Arabia (2007, *n* = 2,574; 2010, *n* = 1,797; 2022, *n* = 5,610). The GYTS uses a two-stage cluster sampling design to select students aged 13–15 from intermediate schools, with a focus on tobacco use trends and related behaviors.

**Results:**

This study revealed a substantial decline in current tobacco product usage, dropping significantly from 15.9% in 2007 to 9.4% in 2022 (*B* = −0.44, *p* = 0.030), and a decrease in exposure to second-hand smoke from 27.9% in 2007 to 18.1% in 2022. However, the proportion of individuals aged 7 years or younger who initiated cigarette smoking rose from 9.1% in 2007 to 17.1% in 2022. There was a 6.4% increase among female youth who thought that smoking made people feel comfortable across this time period (*B* = 0.55, *p* = 0.033), and this population demonstrated a significant decrease in the intention to quit smoking from 65.7 to 52.2% (*B* = −0.895, *p* = 0.010).

**Conclusion:**

Although overall tobacco use decreased, alarming trends, emerged, such as earlier smoking initiation and declining intentions to quit among females. Future interventions should focus on preventing early tobacco use initiation by addressing misconceptions, promoting cessation, and enforcing restrictions on youth access to tobacco products.

## 1 Introduction

### 1.1 Tobacco use in Saudi Arabia

The burden of tobacco use remains a significant global public health challenge, causing over 8 million deaths annually, primarily in low- and middle-income countries (1). In Saudi Arabia, the prevalence of current smoking among adults was approximately 12% in 2013 (2). By 2018, data from the Saudi Food and Drug Authority (SFDA) indicated an increased prevalence of smoking, with 21% of adults reporting current smoking (3). In 2019, the Global Adult Tobacco Survey (GATS) revealed that the prevalence of tobacco use among adults aged 15 years and above in Saudi Arabia reached 19%, with a higher prevalence among men (30%) than among women (4%). The survey also revealed that the average age of daily smoking initiation is 18 years (4). These factors position tobacco control as a critical public health priority in Saudi Arabia, particularly given that smoking is responsible for an estimated 70,000 deaths annually (5).

### 1.2 Youth and tobacco use in Saudi Arabia

Youth is considered the largest segment of the population in Saudi Arabia (6). Maintaining a healthy status for youth is essential to reduce the risk of many preventable chronic diseases, particularly those related to tobacco use, such as hypertension, cardiovascular diseases, and cancers (7). In Saudi Arabia, the prevalence of hypertension is greater than that in other Arab countries (8), and there is a specific increasing trend of cardiovascular disease among youth (9). In addition, the tobacco industry has historically employed aggressive marketing strategies targeting adolescents, referring to them as “replacement smokers” (10, 11). In Saudi Arabia, approximately 15% of adolescents reported using tobacco in 2010 (12). A systematic review of studies published between 2007 and 2018 revealed that the prevalence of tobacco smoking among adolescents in Saudi Arabia ranged from 12.7 to 39.6% (13). These data indicate a wide range of prevalence rates attributable to various sampling methodologies employed across the included studies.

Furthermore, several studies have demonstrated similar variations in cigarette smoking prevalence according to region and sex (2, 12–14). One 2025 article revealed that the prevalence of youth who had ever used e-cigarettes in Saudi Arabia was 14.2% (15). Despite the implementation of educational programs and anti-tobacco campaigns, younger individuals continue to use tobacco (16).

### 1.3 Tobacco control efforts in Saudi Arabia

The Saudi Ministry of Health (MOH) launched a national tobacco control program following Saudi Arabia’s ratification of the World Health Organization (WHO) Framework Convention on Tobacco Control (FCTC) in 2005 (5, 17). Efforts to combat smoking began in the early 2000s. In 2009, the Gulf Cooperation Council (GCC) Standardization Organization (GSO) introduced a technical regulation that included provisions for limits on cigarette emissions, such as tar, nicotine, and carbon monoxide (18). This was followed in 2011 by GSO regulations mandating labeling requirements for tobacco product packaging (19). That same year, the MOH established specialized tobacco cessation clinics to support individuals seeking to quit smoking (12). Between 2012 and 2022, the government adopted more aggressive measures to control tobacco use (20). In 2015, Royal Decree No. 56 introduced the Anti-Smoking Law, a comprehensive framework for tobacco control in Saudi Arabia. The law imposed restrictions on smoking in public places, limited tobacco advertising, promotion, and sponsorship, and set requirements for health warnings on tobacco packaging (21). Additional measures followed, including a substantial 100% increase in tobacco taxes, an expansion of the smoking ban to include public establishments and restaurants, and the implementation of penalties for violations of smoking regulations.

Furthermore, the MOH ensures better access to high-quality treatment and preventive services; thus, it has collaborated with the Ministries of Education, Transportation, Interior Affairs, and the SFDA. The MOH has also launched several anti-smoking campaigns through mass media and social media platforms, aiming to raise awareness about the dangers of smoking (20). To support individuals who wish to quit, various smoking cessation services, including free treatment programs and the “937” Smoking Cessation Help Service hotline, have been established for citizens, residents, and visitors in the Kingdom of Saudi Arabia (22). In 2018, the SFDA issued a circular to tobacco product importers, mandating compliance with plain packaging regulations for tobacco products (20). By 2019, these services were made widely accessible through a network of 274 hospital-based clinics, 321 primary healthcare centers, 890 fixed smoking cessation clinics, 100 mobile cessation clinics, 283 home care services, and 14 specialized hospitals (12).

Although Saudi Arabia was recognized by the World Health Organization (WHO) in 2018 as one of 23 “best practice” countries for its exemplary efforts in providing effective tobacco dependence treatment (23), systematically tracking cigarette smoking prevalence since the implementation of these regulations has remained critically important. Despite increasing concerns about adolescent smoking in Saudi Arabia, comprehensive national-level evidence to fully understand its prevalence and associated factors is lacking.

Monitoring smoking trends over time is essential for informing public health policies and designing effective interventions, enabling the development of targeted strategies to prevent smoking initiation and reduce overall prevalence. Furthermore, these insights are crucial for optimizing resource allocation to address tobacco-related health challenges. The lack of a nationally representative tobacco survey in the past has limited the ability to analyze trends over time. Saudi Arabia conducted two waves of the Global Youth Tobacco Survey (GYTS), a nationally representative survey, in 2007 and 2010; however, these surveys were undertaken prior to the ratification of the FCTC (24). The recent administration of the GYTS by the Saudi MOH in 2022 presents an opportunity to assess tobacco trends in Saudi Arabia and examine whether tobacco-related health risks have changed over time. Such analyses could provide a better estimate of the current and future health burden of tobacco use, including its contribution to chronic diseases and its economic impact on the healthcare system. Therefore, this study investigated trends in tobacco use among youth in Saudi Arabia from 2007 to 2022 and evaluated whether the determinants of tobacco use and control have evolved over time.

## 2 Methodology

### 2.1 Study design and data source

This study employed an observational, cross-sectional design. GYTS data, which are nationally representative, were utilized. The GYTS is a cross-sectional, school-based survey of grade 1–3 intermediate school students (typically aged 13–15 years). In Saudi Arabia, the GYTS was conducted in 2007, 2010, and 2022. Three waves of the GYTS were included in this study. GYTS data from two waves (2007 and 2010) are publicly available,^1^ whereas 2022 GYTS data were obtained with permission from the Ministry of Health in Saudi Arabia (IRB log No: 23–106 M).

### 2.2 Study tool

The GYTS, a component of the Global Tobacco Surveillance System (GTSS), is a global standard for systematically monitoring youth tobacco use and tracking key indicators of tobacco control. The survey uses a standard core questionnaire, sample design, and data collection protocol. The questionnaire is self-administered via scannable paper-based bubble sheets. The questionnaire covers the following topics: tobacco use, cessation, second-hand smoke (SHS), pro- and anti-tobacco advertising and promotion, access to and availability of tobacco products, and knowledge and attitudes regarding tobacco use. Demographic data, including age, sex, income, and residency, were also collected.

### 2.3 Sampling technique

The GYTS employs a global standardized methodology that incorporates a two-stage sample design, where schools are selected with probability proportional to their enrollment size. The classes within the selected schools were chosen randomly, and all the students in the selected classes were eligible to participate in the survey. A two-stage cluster sample design was used to produce a representative sample of the students.

School Level—The first-stage sampling frame included all schools with 1st–3rd-grade intermediate classes of 40 or more students (mainly aged 13–15 years). Schools were selected with a probability proportional to the school enrollment size.

Class Level—The second sampling stage involved systematic, equal-probability sampling (with a random start) of classes from each participating school. All classes in the selected schools were included in the sampling frame. All the students in the selected classes were eligible to participate in the survey.

### 2.4 Study inclusion/exclusion criteria

The GYTS was conducted by the Saudi Arabia MOH in 2007, 2010, and 2022. All GYTS waves were included in this study. Male and female students aged 13–15 years were considered.

### 2.5 Analysis

Various personal characteristics, including demographic, intra- and interpersonal, environmental, and social factors, were analyzed. Additionally, variables related to tobacco control policies, which included smoke-free policies, restrictions on tobacco advertisements, awareness-raising initiatives, and measures to prevent minors from accessing tobacco, among others, were examined. Appendix 1 presents the key questions considered in the study.

Variables similar across the three studies were integrated into a single dataset for analysis. During data management and cleaning, some variables were recoded to standardize them across the 3 years, aiming to simplify the analysis and interpretation. Subsequently, descriptive and linear regression analyses were performed to determine the rate of change over time (independent variable) while controlling for confounding factors, including age, sex, and grade level. The dependent variables across the analyses were categorized into the following domains: (A) tobacco prevalence; (B) age of smoking initiation; (C) exposure to second-hand smoke; (D) exposure to marketing activities; (E) tobacco-related attitudes, perceptions, and knowledge; and (F) intention to quit smoking (Appendix 1). Detailed information is provided regarding the chosen variables within each domain. The analyses were conducted using IBM SPSS Statistics 27 software.

## 3 Results

### 3.1 Study population characteristics

The data from GYTS surveys conducted in 2007 (*n* = 2,574), 2010 (*n* = 1,797), and 2022 (*n* = 5,610) were analyzed. As presented in Table 1, the age distribution across surveys showed slight variation, with the 2022 survey having a greater proportion of 13-year-olds (34.4%) than the 2007 (26.7%) and 2010 (31.7%) surveys. The sex distribution remained relatively balanced across all three surveys, with male participation ranging from 46.9 to 50.9%. Most of the participants were enrolled in the 1st–3rd intermediate grades, with the 2nd intermediate grade consistently representing the largest proportion of students (37.1–40.3%).

**Table 1:** Sociodemographic characteristics of GYTS survey participants in Saudi Arabia, 2007–2022.

Demographic characteristics		2007	2010	2022
		*n* = 2,574 (%)^a^	*n* = 1797 (%)	*n* = 5,610 (%)
Age	13 years old	685 (26.7)	558 (31.7)	1928 (34.4)
	14 years old	943 (36.2)	616 (34.1)	2,140 (37.1)
	15 years old	946 (37.1)	623 (34.2)	1,542 (28.5)
Sex	Male	1,070 (46.9)	788 (49.7)	2,941 (50.9)
	Female	1,353 (53.1)	927 (50.3)	2,669 (49.1)
Grade	1st Intermediate	933 (36.7)	571 (34.1)	1,672 (31.0)
	2nd Intermediate	1,047 (40.3)	683 (36.0)	2,214 (37.1)
	3rd Intermediate	587 (23.0)	523 (29.9)	1,653 (31.9)

^a^Data are presented as absolute counts and weighted percentages.

### 3.2 Trends in tobacco use prevalence and age of smoking initiation

The analysis of tobacco use behaviors revealed several significant trends between 2007 and 2022 (Table 2). The prevalence of current tobacco product use significantly decreased by 6.5% (*B* = −0.44, *p* = 0.030), declining from 15.9% in 2007 to 9.4% in 2022. Similarly, the use of other tobacco products demonstrated a significant reduction of 4.4% (*B* = −0.293, *p* = 0.003), dropping from 11.9 to 7.5% over the study period. Sex-specific analysis revealed that boys experienced a significant decrease in ever-smoking cigarettes, with a 9.6% decrease (*B* = −0.657, *p* = 0.048), resulting in a decline in prevalence from 35.8% in 2007 to 26.2% in 2022. Girls showed no significant trend in ever smoking cigarettes; however, the prevalence increased slightly, albeit not significantly, rising by 5.2% from 16.1% in 2007 to 21.2% in 2022 (*B* = 0.376, *p* = 0.172). Current cigarette smoking rates notably decreased across both genders, dropping by 3.8 percentage points from 6.7% in 2007 to 2.9% in 2022. The intention to initiate smoking among never smokers decreased substantially from 19.2% in 2007 to 7.9% in 2022, a change of 11.3 percentage points. Although findings related to the age of smoking initiation were not significant, they showed concerning trends, with an increase in early initiation (Table 2). The proportion of students who started smoking cigarettes at the age of 7 or younger increased by 8%, from 9.1% in 2007 to 17.1% in 2022. Similar patterns were observed for shisha use, with substantial proportions initiating use at young ages across all survey years.

**Table 2:** Trends in tobacco use prevalence and age of smoking initiation among Saudi adolescents by gender, 2007–2022.

Characteristics of tobacco use			2007	2010	2022	B	*p* value
All							
Prevalence of Tobacco uses		Ever smoked cigarettes	26.1	24.9	23.8	−0.136	0.228
		Currently use any tobacco product	15.9	14.9	9.4	−0.44	0.030*
		Currently smoke cigarettes	6.7	8.9	2.9	−0.324	0.357
		Currently use other tobacco products	11.9	11	7.5	−0.293	0.003*
		Willingness to smoke next year among never smokers	19.2	21.2	7.9	−0.855	0.21
Initiation Age ¶	Cigarettes	7 years old or younger	9.1	13.7	17.1	0.462	0.267
		8 or 9 years old	12.5	8.2	19.5		
		10 or 11 years old	21.4	19.1	18.2		
		12 or 13 years old	32.7	28.2	26.6		
		14 or 15 years old	24.3	30.8	18.7		
	Shisha	7 years old or younger	18.4	14.2	16.8	−0.014	0.966
		8 or 9 years old	13.3	8.9	11.8		
		10 or 11 years old	17	39.4	14.3		
		12 or 13 years old	31.2	16.1	29.1		
		14 or 15 years old	20.1	21.4	27		
Boys							
Prevalence of Tobacco uses		Ever smoked cigarettes	35.8	34.6	26.2	−0.657	0.048*
		Currently use any tobacco product	20.2	21.2	10.6	−0.71	0.175
		Currently smoke cigarettes	10.2	13	3.5	−0.545	0.306
		Currently use other tobacco products	13.3	15.3	8.4	−0.398	0.303
		Willingness to smoke next year among never smokers	20.6	23.8	7.8	−0.99	0.242
Initiation Age ¶	Cigarettes	7 years old or younger	9.7	13	17.5	0.479	0.156
		8 or 9 years old	14.6	7.4	23.6		
		10 or 11 years old	21.4	18.5	18.3		
		12 or 13 years old	33.2	27	25.4		
		14 or 15 years old	21.2	34	15.2		
	Shisha	7 years old or younger	15.9	7.9	16.4	0.226	0.754
		8 or 9 years old	14.4	6.9	13.4		
		10 or 11 years old	17.1	50.8	12.2		
		12 or 13 years old	31.1	10	32		
		14 or 15 years old	21.5	24.5	24.9		
Girls							
Prevalence of Tobacco uses		Ever smoked cigarettes	16.1	15.6	21.2	0.376	0.172
		Currently use any tobacco product	10.7	9.1	8	−0.155	0.280
		Currently smoke cigarettes	2.6	5	2.2	−0.086	0.703
		Currently use other tobacco products	9.4	7.1	6.3	−0.167	0.386
		Willingness to smoke next year among never smokers	16.4	20	7.9	−0.693	0.308
Initiation Age ¶	Cigarettes	7 years old or younger	8.2	13.9	16.6	0.464	0.342
		8 or 9 years old	8.3	10.3	13		
		10 or 11 years old	19.8	22.5	17.9		
		12 or 13 years old	31.7	30.3	28.4		
		14 or 15 years old	32	23	24.1		
	Shisha	7 years old or younger	15.7	27.6	17.3	−0.169	0.867
		8 or 9 years old	11.2	10.8	9.8		
		10 or 11 years old	18.2	19.4	16.7		
		12 or 13 years old	35.3	26.5	25.8		
		14 or 15 years old	19.7	15.6	29.5		

Current tobacco smokers: Smoked cigarettes, shisha, or other types of tobacco anytime during the past 30 days.

Current cigarette smokers: Smoked cigarettes anytime during the past 30 days.

Current smokers of other tobacco: Smoked tobacco other than cigarettes, including shisha, anytime during the past 30 days.

B: regression coefficient, *p* < 0.05 is statistically significant.

*: Significant results.

¶: Age group reference for smoking initiation was 7 years old or younger.

### 3.3 Exposure to secondhand smoke and tobacco marketing activities

Exposure to second-hand smoke tended to decrease across all measured indicators (Table 3). The percentage of students living in homes where others smoke decreased by 9.8%, from 27.9% in 2007 to 18.1% in 2022. Notably, the percentage of boys with one or more parents who smoked significantly decreased by 10.5% from 25.9 to 15.4% (*B* = −0.702, *p* = 0.006). Exposure to smoke outside the home also decreased by 9.7%, from 38.2 to 28.5%, over the study period. Moreover, exposure to tobacco marketing activities such as advertisements and promotions showed a declining trend, with the percentage of students who reported seeing tobacco use in the media decreasing by 35.7%, from 83.8 to 48.1% (Table 3).

**Table 3:** Changes in exposure to second-hand smoke and marketing activities among Saudi adolescents by gender and setting, 2007–2022.

(A) Exposure to second-hand smoke	2007	2010	2022	B	*p* value
All					
Exposed to second-hand smoking inside home	27.9	29.5	18.1	−0.738	0.204
Exposed to second-hand smoking outside home	38.2	37.5	28.5	−0.676	0.080
Having smoking parents (one or both)	24.3	21	16.4	−0.486	0.152
Having smoking friends	7.9	6.3	4.4	−0.212	0.181
Boys					
Exposed to second-hand smoking inside home	28.9	29.9	14.4	−1.06	0.158
Exposed to second-hand smoking outside home	45.1	44.5	27.2	−1.264	0.102
Having smoking parents (one or both)	25.9	23.9	15.4	−0.702	0.006*
Having smoking friends	11.5	6.7	4.9	−0.357	0.376
Girls					
Exposed to second-hand smoking inside home	26.4	28.8	21.9	−0.379	0.344
Exposed to second-hand smoking outside home	31.6	31.1	29.9	−0.110	0.064
Having smoking parents (one or both)	25.7	21.4	17.5	−0.483	0.230
Having smoking friends	3.8	4.8	3.7	−0.031	0.735

(B) Exposure to tobacco marketing activities	2007	2010	2022	B	*p* value
All					
Exposed to tobacco imagery on television, videos, or movies	83.8	81.9	48.1	−2.505	0.091
Exposed to tobacco advertisements or promotions at points of sale	27.4	29.6	19.7	−0.602	0.257
Offered free tobacco product from tobacco representative	7.9	7.4	6.7	−0.074	0.151
Owned items with tobacco brand logo	11.7	12.3	9.3	−0.186	0.242
Boys					
Exposed to tobacco imagery on television, videos, or movies	85.2	82.7	45.8	−2.755	0.085
Exposed to tobacco advertisements or promotions at points of sale	25.8	28.6	19.6	−0.51	0.318
Offered free tobacco product from tobacco representative	9.3	7.7	9	0.017	0.901
Owned items with tobacco brand logo	12.8	11.8	11.5	−0.069	0.404
Girls					
Exposed to tobacco imagery on television, videos, or movies	84.4	82.2	50.4	−2.376	0.084
Exposed to tobacco advertisements or promotions at points of sale	28.9	30.4	20	−0.671	0.206
Offered free tobacco product from tobacco representative	5.3	6.1	4.5	−0.076	0.454
Owned items with tobacco brand logo	9.1	12	6.9	−0.226	0.505

B: regression coefficient, *p* < 0.05 indicates significance, (*): significant results.

### 3.4 Tobacco-related attitudes, perceptions, knowledge, and intention to quit

Perceptions of smoking showed significant changes among girls (Table 4). There was a 6.8% decrease in those who thought that smokers were attractive (*B* = −0.676, *p* = 0.028), whereas there was a 6.4% increase in those who believed that smoking helped people feel more comfortable (*B* = 0.55, *p* = 0.033). The proportion of students taught about the dangers of smoking in class decreased by 21.4%, from 58.8% in 2007 to 37.4% in 2022, whereas exposure to anti-smoking media messages declined by 18.4%, from 61.8 to 43.4%. Further subanalysis of intentions to quit smoking among current tobacco users revealed a significant declining trend among girls, with their quit intention decreasing by 13.5%, from 65.7 to 52.2% (*B* = −0.895, *p* = 0.010). Compared with girls, boys had a higher but declining quit intention rate of 17.4%, decreasing from 77.1 to 59.7% (Table 4).

**Table 4:** Trends in tobacco-related attitudes, perceptions, knowledge and intentions among Saudi adolescents by gender, 2007–2022.

(A) Tobacco-related attitudes, perceptions, and knowledge (all adolescents)	2007	2010	2022	B	*p* value
All					
Thought smoking is an attractive behavior	26.1	21.5	14.1	−0.748	0.127
Thought smoking helps people feel comfortable	15.5	14.7	25.7	0.748	0.163
Thought quitting is difficult	8.1	NA	15	NA	NA
Being taught about the dangers of smoking in school	58.8	42.1	37.4	−1.131	0.412
Exposed to anti-smoking messages in media	61.8	69.7	43.4	−1.502	0.310
Exposed to anti-smoking messages at public events	60.5	72	18.6	−3.267	0.252
Boys					
Thought smoking is an attractive behavior	31.8	25.3	18.2	−0.817	0.196
Thought smoking helps people feel comfortable	14.4	12.2	28	1.024	0.203
Thought quitting is difficult	10.4	NA	13.1	NA	NA
Being taught about the dangers of smoking in school	66.1	45.9	38.6	−1.483	0.381
Exposed to anti-smoking messages in media	65.3	69.4	46.4	−1.448	0.228
Exposed to anti-smoking messages at public events	64.9	73.3	20.9	−3.343	0.216
Girls					
Thought smoking is an attractive behavior	20	17.5	9.7	−0.676	0.028*
Thought smoking helps people feel comfortable	14.9	17	23.3	0.55	0.033*
Thought quitting is difficult	5.6	NA	17	NA	NA
Being taught about the dangers of smoking in school	52.9	38.7	36.1	−0.862	0.454
Exposed to anti-smoking messages in media	58.2	69.4	40.3	−1.545	0.370
Exposed to anti-smoking messages at public events	57.8	70.8	16.5	−3.26	0.268

(B) Intention to quit smoking (current tobacco users)	2007	2010	2022	B	*p* value
All					
Willingness to stop smoking	71.7	64.8	55.8	−0.971	0.164
Boys					
Willingness to stop smoking	77.1	67.2	59.7	−1.007	0.263
Girls					
Willingness to stop smoking	65.7	62.8	52.2	−0.895	0.010*

B: regression coefficient, *p* < 0.05 indicates significance, (*): significant results.

## 4 Discussion

The goal of this study was to assess the prevalence of tobacco use among youth in Saudi Arabia from 2007 to 2022 using data from the GYTS. The findings highlight progress, including a significant decrease in the prevalence of tobacco use among adolescents in intermediate schools, typically aged 13 and 15, particularly in overall and current cigarette smoking rates. However, trends such as an increase in early smoking initiation and persistent exposure to tobacco advertisements and promotions emphasize the need for continued monitoring and the implementation of targeted behavioral public health interventions to combat and prevent the spread and persistence of this issue. These findings may provide valuable insights for enhancing tobacco control efforts and further reducing tobacco-related health risks among Saudi youth. Moreover, our study offers a comprehensive overview of tobacco use trends among youth over time, serving as a critical resource for decision-makers, researchers, and governments seeking to develop effective strategies to address this pressing public health challenge.

Our findings revealed a significant trend in tobacco use, with a marked decrease over the study period. This decline may be attributable to global efforts to combat tobacco use, alongside initiatives implemented by the Saudi government to limit and prevent tobacco consumption across different age groups. These initiatives included increased value-added taxes on tobacco, the launch of anti-smoking campaigns, and the establishment of anti-smoking clinics, including the introduction of a mobile app. These comprehensive measures reflect Saudi Arabia’s commitment to reducing tobacco use and its associated health risks among its population (5, 25). Furthermore, our study may be among the first to measure the influence of these initiatives on tobacco prevalence in Saudi Arabia.

This study highlights a significant decrease in exposure to second-hand smoke over time. A previous study by Al-Zalabani et al. (26) highlighted a concerning prevalence of second-hand smoke exposure in public places and homes, particularly among adolescents, indicating an urgent need for increased awareness regarding the harmful effects of passive smoking. The reduction in exposure to second-hand smoke observed in our study can be attributable to the implementation of comprehensive tobacco control initiatives and policies aimed at mitigating factors enhancing tobacco use (5). This reduction has significant implications for the overall decrease in tobacco use among youth in Saudi Arabia, as subjective norms reflecting the collective attitudes of smokers in public have changed, leading to a lower risk of tobacco use initiation (27). Collectively, these measures demonstrate the effectiveness of strategic public health interventions in creating a healthier, smoke-free environment.

The findings of this study revealed a noticeable shift in students’ perceptions of tobacco use. Although the proportion of students who associated smoking with attractiveness decreased over time, the proportion of those who perceived smoking as a positive behavior in social contexts increased, particularly among girls. In addition to our findings, previous studies have demonstrated a positive attitude toward smoking (28, 29), suggesting a concerning change in the social appeal of smoking among youth. While the findings of this study revealed a decreasing trend in exposure to tobacco advertisements and promotions, the persistence of the belief that smoking facilitates comfort may be influenced by media portrayals that depict tobacco use as a means of relaxation and social acceptance (30). Therefore, future research should consider the sources from which misconceptions about tobacco use arise when developing tobacco control interventions targeting youth.

Furthermore, our study revealed a concerning decline in the proportion of individuals receiving formal education on the dangers of smoking and exposure to anti-smoking media campaigns. Despite the implementation of strong public health policies, such as graphic warning labels on cigarette packaging, comprehensive bans on tobacco advertising, and the rise of social media platforms promoting healthier lifestyles in general (5, 31), our findings indicate a lag in health awareness and education regarding the harmful effects of tobacco use. Although official reports document ongoing anti-tobacco educational campaigns (32), the results of this study suggest a lack of visibility for anti-tobacco messages. This interpretation is further supported by previous research, which indicates that more effective tobacco control educational campaigns rely on several key factors, including reach, the informational content of messages, message intensity, delivery mode, and duration (33).

The declining trend observed in our study is alarming, as such interventions have been critical in reinforcing knowledge about the risk of tobacco use, fostering community awareness, and ultimately reducing tobacco use among young adults (34, 35). Future efforts should focus on creating effective and culturally tailored anti-tobacco educational campaigns that resonate with the target audience. Investing in anti-tobacco educational interventions within schools and universities, as well as enhancing anti-smoking media campaigns, is crucial for addressing the issue of tobacco use among youth. However, it is equally important to accompany knowledge dissemination with the development of social competence skills that empower youth to resist tobacco product offers (36). Strengthening these efforts can help counter the influence of smoking and reinforce the importance of limiting tobacco use among youth.

Moreover, the intention to quit smoking demonstrated a substantial declining trend, particularly among girls. Several factors may influence the intention to quit smoking among youth, including sociodemographic, social, behavioral, and environmental factors (37, 38). Further investigation into the factors associated with the lack of intention to quit smoking among youth, especially females, is needed to promote smoking cessation. In addition, the findings of this study highlight a critical area for intervention, emphasizing the need for tailored cessation support and targeted public health strategies to address the decreasing motivation to quit smoking. The age of smoking initiation showed troubling trends, with a notable proportion of students reporting their first experience with tobacco at 7 years of age or younger. This increasing trend may be attributable to easy access to tobacco products through social or commercial sources (39), as the GYTS fact sheet mentioned that 51.7% of youth were not refused the purchase of tobacco products due to their age (40). Consequently, there is an urgent need to implement targeted prevention strategies and actively enforce existing tobacco control measures to address early tobacco experimentation and use among youth.

### 4.1 Limitations

This study has several limitations. First, the use of secondary data from the GYTS may impact reliability, as inconsistencies in survey administration and variations in sampling methods may have existed across the years. Second, relying on self-reported data may imply the possibility of recall and social desirability biases, which could influence the accuracy of the reported prevalence of tobacco use and related behaviors. Third, owing to the cross-sectional nature of the study design, establishing temporal relationships between variables was not possible. Incorporating longitudinal follow-up data could help in assessing changes in tobacco trends and enhance the development of causal relationships. Fourth, the study analysis focused on adolescents aged 13–15 years, which limits the generalizability of the findings to other age groups, such as young adults. Thus, expanding the age range of participants to include young adults would enhance the generalizability of the findings and help in capturing tobacco use beyond the cohort of 13–15 years. Finally, variations in the implementation of tobacco control policies over time may not be fully captured by the study design, limiting the ability to attribute changes in tobacco use directly to specific interventions.

## 5 Conclusion

The findings from this study show a decline in the prevalence of tobacco use among Saudi adolescents between 2007 and 2022, highlighting progress in tobacco control efforts within the country. However, trends such as early smoking initiation, limited exposure to anti-tobacco education, and misconceptions related to tobacco use underscore the urgent need for ongoing and targeted public health interventions. Understanding the trends of tobacco use among youth over time in Saudi Arabia and assessing changes in factors associated with tobacco use could help policymakers initiate targeted tobacco control initiatives and allocate resources efficiently. Addressing gaps in anti-smoking education, limiting access to tobacco products, and promoting cessation support for youth remain critical priorities. Our study encourages future initiatives to maintain progress and achieve long-term reductions in tobacco-related morbidity and mortality in Saudi Arabia.

## Data Availability

Publicly available datasets were analyzed in this study. This data can be found here: https://www.cdc.gov/tobacco/global/.

## Footnotes

^1^ https://www.cdc.gov/tobacco/global/gtss/
