# Supplementary material for: Tobacco use trends among youth in Saudi Arabia: 2007–2022
Source: Front Public Health. 2025 Jul 2;13:1608394. doi: 10.3389/fpubh.2025.1608394 (PMC12263556; doi:10.3389/fpubh.2025.1608394)
Supplement: Supplementary file 1 [file Table_1.pdf]

Appendix. 1. Global Youth Tobacco Survey (GYTS) Variables Considered in the Study Analysis

Domains		Variables	Questions and their response
Personal characteristics	Demographics	Age	**How old are you?**<br>a. 11 years old or younger<br>b. 12 years old<br>c. 13 years old<br>d. 14 years old<br>e. 15 years old<br>f. 16 years old<br>g. 17 years old or older
		Gender	**What is your sex?**<br>a. Male<br>b. Female
		Grade Level	**In what grade are you?**<br>a. 1st Intermediate<br>b. 2nd Intermediate<br>c. 3rd Intermediate
Monitoring tobacco use	Prevalence in tobacco use and age of smoking initiation	Ever smoked cigarettes	**Have you ever tried cigarette smoking, even one or two puffs?**<br>a. Yes<br>b. No
		Currently use any tobacco product	**During the past 30 days, on how many days did you smoke cigarette, shisha, or other types of tobacco?**<br>a. 0 days<br>b. 1 or 2 days<br>c. 3 to 5 days<br>d. 6 to 9 days<br>e. 10 to 19 days<br>f. 20 to 29 days<br>g. All 30 days
		Currently smoke cigarettes	**During the past 30 days, on how many days did you smoke cigarette?**<br>a. 0 days<br>b. 1 or 2 days<br>c. 3 to 5 days<br>d. 6 to 9 days<br>e. 10 to 19 days<br>f. 20 to 29 days<br>g. All 30 days
		Currently use other tobacco products	**During the past 30 days, on how many days did you smoke any type of tobacco other than cigarettes?**<br>a. 0 days<br>b. 1 or 2 days<br>c. 3 to 5 days<br>d. 6 to 9 days<br>e. 10 to 19 days<br>f. 20 to 29 days<br>g. All 30 days
		Willingness to smoke next year	**At any time during the next 12 months, do you think you will use any form of tobacco?**<br>a. Definitely not

		among never smokers	b. Probably not<br>c. Probably yes<br>d. Definitely yes
		Initiation Age	**How old were you when you first tried a cigarette/shisha?**<br>a. I have never tried smoking a cigarette<br>b. 7 years old or younger<br>c. 8 or 9 years old<br>d. 10 or 11 years old<br>e. 12 or 13 years old<br>f. 14 or 15 years old<br>g. 16 years old or older
Social and environmental factors<br>Tobacco control policy: smoke-free policy	Exposure to secondhand smoking	Exposed to secondhand smoking inside home	**During the past 7 days, on how many days has anyone smoked inside your home, in your presence?**<br>a. 0 days<br>b. 1 to 2 days<br>c. 3 to 4 days<br>d. 5 to 6 days<br>e. 7 days
		Exposed to secondhand smoking outside home	**During the past 7 days, on how many days has anyone smoked in your presence, outside your home: inside any enclosed public place, other than your home (Such as schools, shops, restaurants, shopping malls, cinemas, theaters, cafes, rest houses, government buildings)/ outdoor public place (such as playgrounds, gardens, and parks, outdoor seating in restaurants or cafes, building entrances, resorts, beaches)?**<br>a. 0 days<br>b. 1 to 2 days<br>c. 3 to 4 days<br>d. 5 to 6 days<br>e. 7 days
		Having smoking parents (one or both)	**Do your parents smoke tobacco?**<br>a. None<br>b. Both<br>c. Father only<br>d. Mother only<br>e. I Don't know
		Having smoking friends	**Do any of your closest friends smoke tobacco?**<br>a. None of them<br>b. Some of them<br>c. Most of them<br>d. All of them
Environmental factors<br>Tobacco control policy: Restrictions on tobacco advertisements and [illegible]	Exposure to marketing activities	Exposed to tobacco imagery on television, videos, or movies	**During the past 30 days, did you see any people using tobacco when you watched TV, videos, or movies?**<br>a. I did not watch TV, videos, or movies in the past 30 days<br>b. Yes<br>c. No
		Exposed to tobacco advertisements or promotions at points of sale	**During the past 30 days, did you see any advertisements or promotions for tobacco products at points of sale (such as Stores, shops, websites)?**<br>a. I did not visit any points of sale in the past 30 days<br>b. Yes<br>c. No
		Offered free tobacco product from tobacco representative	**Has a person working for a tobacco company ever offered you a free tobacco product?**<br>a. Yes<br>b. No

		Owned items with tobacco brand logo	**Do you have something (for example, t-shirt, pen, backpack) with a tobacco product brand logo on it?**<br>a. Yes<br>b. No
Intrapersonal and environmental factors<br>Tobacco control policy: awareness and education about the harmful effect of tobacco	Tobacco-related attitude, perception, and knowledge	Thought smoking is an attractive behavior	**Do you think smoking tobacco makes young people look more or less attractive?**<br>a. More attractive<br>b. Less attractive<br>c. No difference from non-smokers
		Thought smoking helps people feel comfortable	**Do you think smoking tobacco helps people feel more comfortable or less comfortable at celebrations, parties, or other social gatherings?**<br>a. More comfortable<br>b. Less comfortable<br>c. No difference whether smoking or not
		Thought quitting is difficult	**Once someone has started smoking tobacco, do you think it would be difficult for them to quit?**<br>a. Definitely, quitting will not be hard<br>b. Probably, quitting will not be hard<br>c. Probably, quitting will be hard<br>d. Definitely, quitting will be hard
		Being taught about the dangers of smoking in school	**During the past 12 months, were you taught in any of your classes about the dangers of tobacco use?**<br>a. Yes<br>b. No<br>c. I don't know
		Exposed to anti-smoking messages in media	**During the past 30 days, did you see or hear any anti-tobacco media messages on television, radio, internet, social media, billboards, posters, newspapers, magazines, or movies?**<br>a. Yes<br>b. No
		Exposed to anti-smoking messages in public events	**During the past 30 days, did you see or hear any anti-tobacco messages at sports events, fairs, concerts, community events, or social gatherings?**<br>a. I did not go to sports events, fairs, concerts, community events, or social gatherings in the past 30 days<br>b. Yes<br>c. No
	Intention to quit smoking	Willingness to stop smoking	**Do you want to stop smoking now?**<br>a. I have never smoked<br>b. I don't smoke now<br>c. Yes, I want to stop<br>d. No, I don't want to stop

Note: The variables above are found in three GYTS surveys: 2007, 2010, and 2022. Nevertheless, the phrasing of the questions and responses varied across the years. Expert reviewers examined the questions and their responses to ensure consistency, validity, and harmonization, guaranteeing that they represented identical questions over the three-year period, despite being worded differently. In preparation for data analysis, several variables were recoded to simplify the analysis and interpretation.
